# Evaluation of Mpox Knowledge, Stigma, and Willingness to Vaccinate for Mpox: Cross-Sectional Web-Based Survey Among Sexual and Gender Minorities

**DOI:** 10.2196/46489

**Published:** 2023-07-17

**Authors:** Thiago Silva Torres, Mayara Secco Torres Silva, Carolina Coutinho, Brenda Hoagland, Emilia Moreira Jalil, Sandra Wagner Cardoso, Julio Moreira, Monica Avelar Magalhaes, Paula Mendes Luz, Valdilea G Veloso, Beatriz Grinsztejn

**Affiliations:** 1 Instituto Nacional de Infectologia Evandro Chagas Fundação Oswaldo Cruz Rio de Janeiro Brazil; 2 Grupo Arco-Íris Rio de Janeiro Brazil; 3 Instituto de Comunicação e Informação Científica e Tecnológica em Saúde Fundação Oswaldo Cruz (ICICT-Fiocruz) Rio de Janeiro Brazil

**Keywords:** sexual and gender minorities, mpox, Brazil, Latin America and Caribbean, community, LGBT, gay, lesbian, minority, minorities, survey, emergency, stigma, discrimination, Latin America, sociodemographic, behavioral, diagnosis, application, social media, infection, gender, MSM, monkeypox, infectious disease, awareness, patient education, health knowledge, patient knowledge, knowledge translation, access to care, viral

## Abstract

**Background:**

The 2022 multicountry mpox outbreak positioned the condition as a public health emergency of international concern. By May 2023, Brazil ranked second globally in the cumulative number of mpox cases and deaths. The higher incidence of mpox among gay and other men who have sex with men in the current mpox outbreak deepens the stigma and discrimination against sexual and gender minorities (SGM). This might worsen the structural barriers impacting access to health services, which ultimately leads to undertesting and underreporting of cases. There are no data available on mpox knowledge and stigma in Latin America.

**Objective:**

We aimed to evaluate mpox knowledge, stigma, and willingness to vaccinate for mpox among SGM, and to describe sociodemographic and behavioral characteristics according to self-reported mpox diagnosis.

**Methods:**

A cross-sectional, internet-based survey was conducted in a convenience sample of adults (aged >18 years) living in Brazil recruited through advertisements on dating apps, social media, referral institutions for infectious diseases websites, and mass media (October-November 2022). We compared participants’ characteristics according to self-reported mpox diagnosis using chi-square test or Fisher exact test for qualitative variables and Kruskal-Wallis test for quantitative variables.

**Results:**

We enrolled 6236 participants: 5685 (91.2%) were cisgender men; 6032 (96.7%) were gay, bisexual, or pansexual; 3877 (62.2%) were White; 4902 (78.7%) had tertiary education; and 4070 (65.2%) reported low or middle income. Most participants (n=5258, 84.4%) agreed or strongly agreed that “LGBTQIA+ individuals are being discriminated and stigmatized due to mpox.” Mpox awareness was 96.9% (n=6044), and 5008 (95.1%) were willing to get vaccinated for mpox. Overall, 324 (5.2%) reported an mpox diagnosis. Among these, 318 (98.1%) reported lesions, 178 (56%) local pain, and 316 (99.4%) sought health care. Among participants not reporting a diagnosis, 288 (4.9%) had a suspicious lesion, but only 158 (54.9%) of these had sought health care. Compared to participants with no diagnosis, those reporting an mpox diagnosis were younger (*P*<.001), reported more sex partners (*P*<.001), and changes in sexual behavior after mpox onset (*P*=.002). Moreover, participants diagnosed with mpox reported more frequently being tested for HIV in the prior 3 months (*P*<.001), living with HIV (*P*<.001), currently using HIV pre-exposure prophylaxis (*P*<.001), and previous sexually transmitted infection diagnosis (*P*<.001).

**Conclusions:**

Our results point to high mpox knowledge and willingness to vaccinate among SGM in Brazil. Participants self-reporting mpox diagnosis more frequently reported to be living with HIV, STI diagnosis, and current pre-exposure prophylaxis use, highlighting the importance of an mpox assessment that includes comprehensive sexual health screenings. Efforts to decrease stigma related to mpox among SGM are necessary to avoid mpox underdiagnosis.

## Introduction

The 2022 multicountry mpox outbreak positioned the condition as a public health emergency of international concern, as declared by the World Health Organization on July 23, 2022 [1]. Previously, mpox had been barely identified outside specific African countries with a known endemic epidemiology [2]. The first cases in nonendemic countries were announced in the United Kingdom, in May 2022, with over 87,000 confirmed mpox cases worldwide by May 23, 2023 [3,4]. The outbreak has disproportionally burdened vulnerable groups, especially gay, bisexual, and other cisgender men who have sex with men (MSM) [2,5]. Brazil ranked second globally in the cumulative number of mpox cases and deaths, with 10,941 confirmed diagnoses and 16 deaths as of May 23, 2023 [4]. Confirmed mpox cases in Brazil mostly occurred among cisgender MSM, with a potential role of sexual contact in the transmission dynamics [6-8].

Unfortunately, the higher incidence of mpox among vulnerable groups in the current mpox outbreak deepens the stigma and discrimination against MSM and other sexual and gender minorities (SGM) and might worsen the structural barriers impacting access to health services, which ultimately leads to undertesting and underreporting of cases [9-12]. Furthermore, the shame and fear related to societal judgment about individuals’ sexuality combined with the need for prolonged self-isolation have a significant impact on a person’s professional and daily life, thus undermining the mental health of the most vulnerable [13]. Moreover, this phenomenon might not be restricted to the current outbreak. Mpox-related stigma against SGM could be underestimated in countries with endemic mpox, as many of these still criminalize same-sex relationships, and data on cases among MSM might be inaccurate [14].

Efforts are in course to mitigate the impact of an mpox outbreak on the most vulnerable groups, and a stigma-free message that embraces sexual and gender-diverse people is key to establishing strong links connecting health services to the community. For this purpose, it is essential to analyze the knowledge among vulnerable populations about mpox so that communication and health actions can be tailored accordingly. A cross-sectional survey among 1932 respondents in the United Kingdom showed that, despite a vaccine acceptability rate as high as 96%, only 29% would seek a health service in case of mpox symptoms [15]. In the United States, 50% of MSM who responded to a web-based survey reported a 50% reduction in the number of sex partners, 1-time sexual encounters, group sex, and sex with a partner they met on a dating app or at a sex venue after the onset of the current mpox outbreak, although there was no change in condom use [16].

There are no data available on mpox knowledge in Latin America. This is the first survey in Brazil aiming to evaluate mpox knowledge, stigma, and willingness to vaccinate for mpox among SGM, and to describe sociodemographic and behavioral characteristics of study participants according to self-reported mpox diagnosis.

## Methods

### Study Design

This was a cross-sectional internet-based survey conducted in a convenience sample of adults (aged ≥18 years) living in Brazil, who were recruited through advertisements on dating apps (Grindr and Scruff), social media (Facebook and Instagram), referral institutions for infectious diseases websites or social media (Instituto Nacional de Infectologia Evandro Chagas and Fundação Oswaldo Cruz), radio, and mass media from October to November 2022. Banners in Scruff and Grindr and boosted posts on Facebook and Instagram, as in prior work [17,18], were used to recruit individuals to complete a voluntary open survey in Portuguese, with all items including a nonresponse option. The survey was programmed on Alchemer. Respondents were able to change or review answers and did not receive any compensation. Usability and technical functionality in different personal computers and mobile phone operating systems were confirmed before survey administration. We excluded respondents who did not reach the end of the survey and those who incorrectly answered any of the 5 attention questions [19]. Only 1 response per IP address was allowed.

### Variables

#### Sociodemographic

Recruitment was categorized in Grindr, Scruff, Instagram, Facebook, and others (eg, WhatsApp and other websites). Age at the time of the survey was categorized in 4 intervals: 18-24; 25-30, 31-40, and >40 years. Gender categories were cisgender man, transgender man, cisgender woman, transgender woman, nonbinary or gender fluid, queer, and not declared. Sexual orientation categories were lesbian, gay, bisexual, pansexual, heterosexual, or other (eg, asexual and demisexual). Race was categorized as Asian (Japanese, Chinese, Korean, and among others), Black, Indigenous, Pardo (mixed race), White, or not declared. For schooling, the highest degree attained is considered (primary, secondary, or tertiary). Income reporting was based on the monthly income of the household in Brazilian minimum wages (MWs): low (up to 2 MW), middle (>2-6 MW), and high (>6 MW), in accordance with previous studies [18,20]. In 2022, MW per month was BRL 1212 (US $230). Participants were also asked whether they received any social support (eg, conditional cash transfers such as Auxílio Brasil) and the number of persons living in the same house (median, IQR). Brazilian regions were defined according to the Brazilian administrative division: North (7 states), Northeast (9 states), Central-west (3 states and Federal District), South (3 states), and Southeast (4 states). The state region was stratified into capital, metropolitan area, and other cities.

#### Mpox Awareness, Knowledge, Symptoms, and Contact

We defined mpox awareness as a positive answer to the question: “Have you ever heard about mpox?” Knowledge of mpox lesions was assessed with the question: “Do you know, or have you seen pictures of suspected mpox wounds/lesions?” Participants answered where they obtained information about mpox (eg, television, radio, Grindr, Hornet, Scruff, Google, other) and if they would follow social distancing if diagnosed with mpox (yes or no).

The survey also included questions about the participant’s health as it relates to any suspicious lesions related to mpox since the onset of mpox outbreak in Brazil (June 2022) (yes or no), location of the potential lesions, number of legions per location, if attended health facility to investigate lesions (yes or no), if lesions were painful, and graduation of pain from 0 (no pain) to 100 (worst pain). Participants were also asked if they had symptoms possibly related to mpox (predetermined list) since June 2022, and in case of any symptoms, if they attended a health facility to investigate them (yes or no).

Participants were asked about contact (sexual or nonsexual) with persons with suspected or confirmed mpox diagnosis, if traveled within Brazil or abroad since June 2022. Finally, for those self-reporting an mpox diagnosis, we inquired if they adhered to social distancing after receiving the diagnosis.

#### Sexual Behavior

Participants answered questions about their sexual behavior since June 2022, including gender of sex partners, number of sex partners (stratified in 0, 1, 2-5, 6-10, and >10), steady partners (yes or no and if only 1 steady partner), sex practices (insertive or receptive vaginal sex, anal sex, and oral sex), condomless sex for these sex practices, transactional sex, and frequency in sex venues (yes or no). Participants were also questioned about the changes in sexual behavior due to the mpox outbreak (“Have you changed your sexual behavior since mpox cases started in Brazil [June 2022]?”). If yes, they were asked about risk reduction measures adopted from a predetermined list, including the reduced number of sex partners and avoided sex parties.

#### Substance Use and Chemsex

Participants answered questions on substance use since June 2022. Binge drinking was evaluated with the question “Since June 2022, did you drink 5 or more drinks in a couple of hours?” (yes or no) [21]. Participants answered questions about illicit drug use from a predetermined list and an open field option for other substances, later dichotomized into yes or no. Individuals who reported binge drinking or any illicit drug use were inquired about use before or during sex (“binge drinking before or during sex” and “chemsex”) [21,22].

### HIV Testing, Treatment, and Prevention, and Other Sexually Transmitted Infections

HIV self-reported status was derived from the question “Have you ever had an HIV test?” with potential responses positive, negative, or never (“unknown”). Participants were asked when the last HIV test was (≤3, >3-6, and >6 months). People living with HIV answered questions about the time since the diagnosis (≤6, >6-12, and >12 months), about antiretroviral therapy (ART) use, ART adherence using a validated scale [23], and if they currently had an undetectable HIV viral load (yes or no). Participants self-reporting negative or unknown status were asked about HIV pre-exposure prophylaxis (PrEP) use (never, current, or past) and adherence (rated from 0 [missed all doses in past 30 days] to 100 [no missing dose]). We dichotomized complete PrEP adherence (rating=100) into yes or no.

Participants were asked about syphilis, gonorrhea, and chlamydia diagnoses since June 2022 (yes or no), hepatitis B vaccination (no, 1-2 doses, 3 doses, and did not remember), and ever tested positive for hepatitis C (yes or no).

### Adherence to Vaccination Campaigns, and Willingness to Use Vaccines and Treatment for Mpox

Participants responded to questions regarding participation in vaccination campaigns (always, sometimes, or never) and whether they had been vaccinated for COVID-19 and mpox. We used a 4-point Likert scale to access the willingness to use an mpox preventive vaccine, which was defined as responding highly likely to the question: “Would you vaccinate against mpox?” following other studies evaluating the willingness to use biomedical technologies [24,25]. We also accessed willingness to participate in research studies to evaluate: (1) mpox preventive vaccine; (2) mpox preventive drug; and (3) mpox therapeutic drug.

### Internalized Lesbian, Gay, Bisexual, Transgender, Queer, Intersex, Asexual, Plus Phobia, Stigma, and Discrimination

The Reactions to Homosexuality Scale (RHS), originally developed to measure internalized homonegativity among MSM, includes 7 items measured on a 7-point Likert scale from strongly disagree to strongly agree, with total scores ranging from 0 to 42 [18,26]. For this analysis, we adapted the scale to allow completion by all SGM, as described in Table S1 in Multimedia Appendix 1. Higher RHS scores indicate higher internalized lesbian, gay, bisexual, transgender, queer, intersex, asexual, plus (LGBTQIA+) phobia. We also asked participants to rate mpox stigma toward LGBTQIA+ people on a 7-point Likert scale from strongly disagree to strongly agree: “LGBTQIA+ individuals are being discriminated and stigmatized due to mpox.”

### Main Outcome

Our main outcome was self-reported mpox diagnosis, defined as a positive answer to the question: “Have you been diagnosed with mpox by a health professional?”

### Spatial Analysis

We performed an exploratory analysis based on the kernel estimator, with adaptive radius and Gaussian function, to obtain an overview of the spatial distribution of the points corresponding to individuals with confirmed and nonconfirmed mpox diagnosis and identifying potential outbreaks of occurrence. The zip codes were considered for georeferencing.

### Statistical Analysis

Continuous variables were described by their median and IQR and mean and SD. We compared sociodemographic, behavior, and clinical characteristics according to self-reported mpox diagnosis (yes vs no) using a chi-square test or Fisher exact test for qualitative variables and a Kruskal-Wallis test for quantitative variables. All analyses were performed using R software (version 4.2.2; R Foundation for Statistical Computing).

### Ethics Approval

This study received approval from the institutional review board at the Instituto Nacional de Infectologia Evandro Chagas, Fundação Oswaldo Cruz (#CAAE 61290422.0.0000.5262). All study participants provided electronic informed consent before survey initiation.

## Results

**Figure 1:**
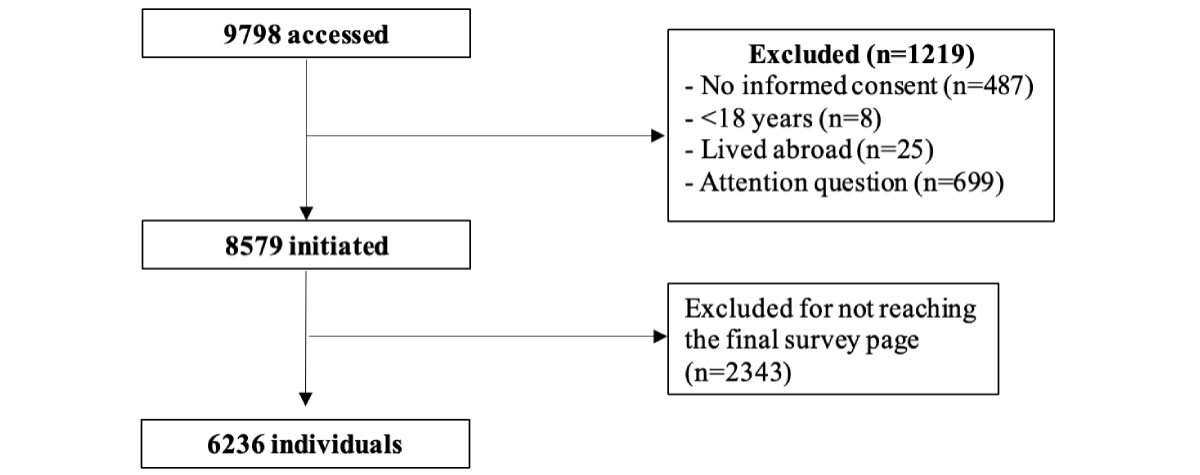
Study flowchart.

A total of 9798 individuals accessed the survey and 1219 (12.4%) did not meet the inclusion criteria (Figure 1). Of the 8579 individuals who initiated the survey, 6236 (72.7%) reached the final survey page and were included in this study. Recruitment mostly occurred on Instagram (n=2331, 37.4%), followed by Scruff (n=1561, 25.0%), Grindr (n=1246, 20.0%), and Facebook (n=692, 11.1%) (Table 1). Median age was 36 (IQR 31-44) years; 91.2% (n=5685) were cisgender men, 83.3% (n=5197) gay, 62.2% (n=3877) White, 78.7% (n=4902) had completed tertiary education, 41.2% (n=2571) reported middle income, 68.3% (n=4259) were from the Southeast region of Brazil, and 67.6% (n=4217) were living in a state capital city.

**Table 1:** Sociodemographic characteristics of the study population according to self-reported mpox diagnosis.

Characteristics		No (n=5912)	Yes (n=324)	Total (N=6236)	*P* value	
**Recruitment, n (%)**						.009
	Instagram	2180 (36.9)	151 (46.6)	2331 (37.4)		
	Scruff	1499 (25.4)	62 (19.1)	1561 (25.0)		
	Grindr	1187 (20.1)	59 (18.2)	1246 (20.0)		
	Facebook	658 (11.1)	34 (10.5)	692 (11.1)		
	Other	387 (6.5)	18 (5.6)	405 (6.5)		
**Age (years)**						
	Median (IQR)	37 (31-44)	34 (30-39.2)	36 (31-44)	<.001	
	18-24, n (%)	363 (6.1)	10 (3.1)	373 (6)	<.001	
	25-30, n (%)	1071 (18.1)	77 (23.8)	1148 (18.4)		
	31-40, n (%)	2369 (40.1)	171 (52.8)	2540 (40.7)		
	>40, n (%)	2109 (35.7)	66 (20.4)	2175 (34.9)		
**Gender, n (%)**						.02
	Cisgender man	5371 (90.8)	314 (96.9)	5685 (91.2)		
	Cisgender woman	245 (4.1)	2 (0.6)	247 (4.0)		
	Queer	156 (2.6)	6 (1.9)	162 (2.6)		
	Nonbinary or gender diverse	89 (1.5)	1 (0.3)	90 (1.4)		
	Transgender man	15 (0.3)	0 (0)	15 (0.2)		
	Transgender woman	13 (0.2)	0 (0)	13 (0.2)		
	Travesti^a^	2 (0)	0 (0)	2 (0)		
	Not declared	21 (0.4)	1 (0.3)	22 (0.4)		
**Sexual orientation, n (%)**						<.001
	Gay	4905 (83.0)	292 (90.1)	5197 (83.3)		
	Bisexual	509 (8.6)	19 (5.9)	528 (8.5)		
	Pansexual	305 (5.2)	2 (0.6)	307 (4.9)		
	Heterosexual	139 (2.4)	11 (3.4)	150 (2.4)		
	Lesbian	19 (0.3)	0 (0)	19 (0.3)		
	Other	35 (0.6)	0 (0)	35 (0.6)		
**Race, n (%)**						.008
	Asian	87 (1.5)	1 (0.3)	88 (1.4)		
	Black	611 (10.3)	52 (16.0)	663 (10.6)		
	Indigenous	30 (0.5)	3 (0.9)	33 (0.5)		
	Pardo	1443 (24.4)	79 (24.4)	1522 (24.4)		
	White	3689 (62.4)	188 (58.0)	3877 (62.2)		
	Not declared	52 (0.9)	1 (0.3)	53 (0.8)		
**Education, n (%)**						.76
	Primary	108 (1.8)	5 (1.5)	113 (1.8)		
	Secondary	1157 (19.6)	59 (18.2)	1216 (19.5)		
	Tertiary	4642 (78.6)	260 (80.2)	4902 (78.7)		
**Income, n (%)**						.08
	Low	1425 (24.1)	74 (22.8)	1499 (24.0)		
	Middle	2420 (40.9)	151 (46.6)	2571 (41.2)		
	High	1886 (31.9)	95 (29.3)	1981 (31.8)		
	Did not want to answer	181 (3.1)	4 (1.2)	185 (3.0)		
**Social support, n (%)**						.47
	No	5510 (93.2)	302 (93.2)	5812 (93.2)		
	Yes	348 (5.9)	17 (5.2)	365 (5.9)		
	Did not want to answer	54 (0.9)	5 (1.5)	59 (0.9)		
Persons living in the same house (n), median (IQR)		2 (1-3)	2 (1-3)	2 (1-3)	.13	
**Brazilian region, n (%)**						.02
	North	134 (2.3)	3 (0.9)	137 (2.2)		
	Northeast	709 (12.0)	29 (9.0)	738 (11.8)		
	Central-west	411 (7.0)	36 (11.1)	447 (7.2)		
	Southeast	4038 (68.3)	221 (68.2)	4259 (68.3)		
	South	620 (10.5)	35 (10.8)	655 (10.5)		
**State region, n (%)**						.003
	Capital	3978 (67.3)	239 (73.8)	4217 (67.6)		
	Metropolitan area	929 (15.7)	53 (16.4)	982 (15.7)		
	Other cities	1005 (17.0)	32 (9.9)	1037 (16.6)		

^a^Female gender construction, which is identified in social, family, cultural, and interpersonal life through this identity.

Most participants had heard of mpox (n=6044, 96.9%), reported knowing how mpox lesions appear (n=5298, 85.0%) (Table 2), and that they would follow social distancing if diagnosed with mpox (n=6051, 97.0%). Information about mpox was mostly obtained on the internet (n=4793, 76.9%) and television (n=4519, 72.5%) (Table S2 in Multimedia Appendix 1).

**Figure 2:**
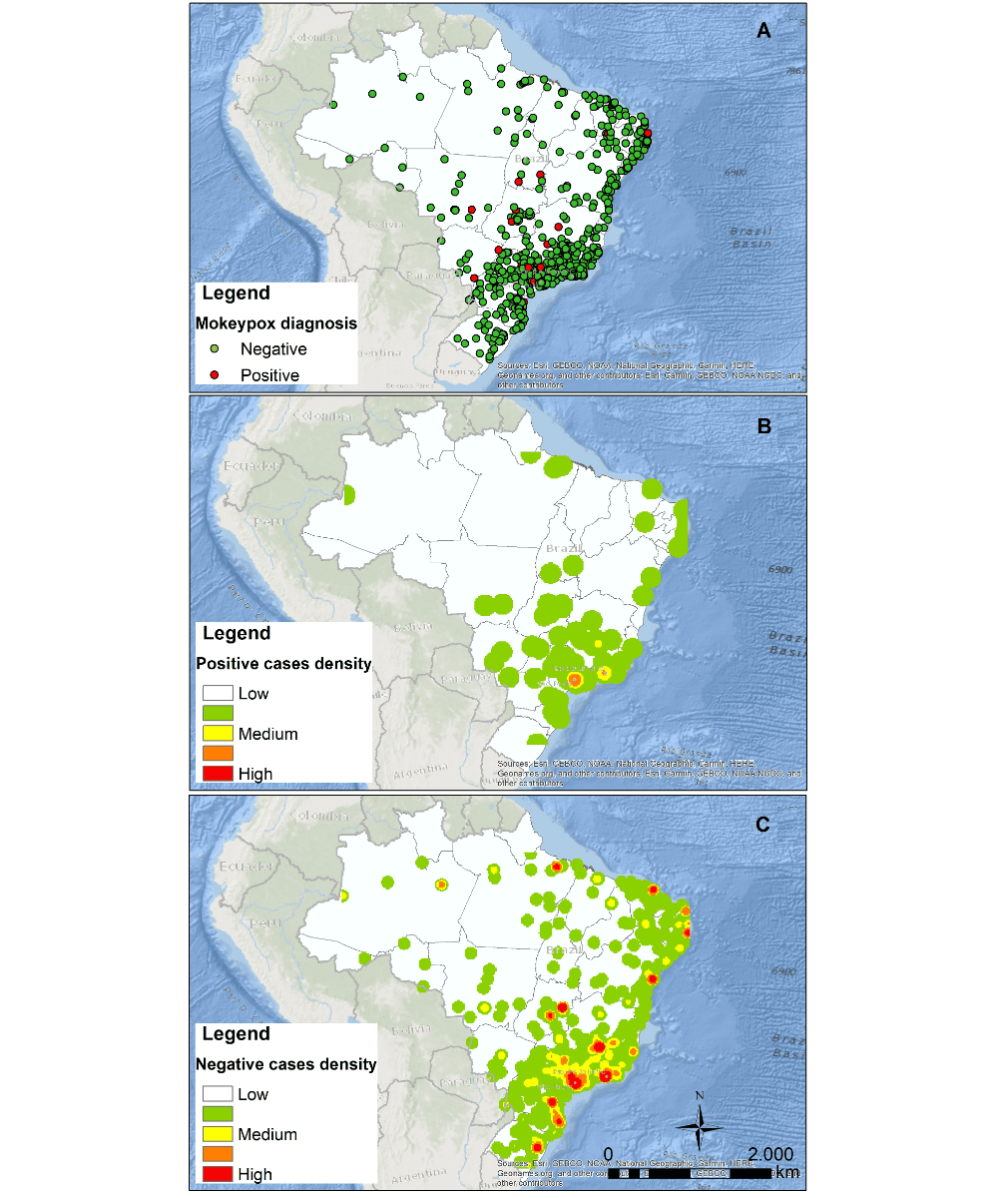
Spatial distribution of individuals with negative and positive self-reported mpox diagnosis.

Overall, 324 (5.6%) individuals reported an mpox diagnosis; 44.4% (n=144) of mpox cases were from São Paulo state, followed by 15.7% (n=51) in Rio de Janeiro (Table S3 in Multimedia Appendix 1). Hotspots of participants reporting an mpox diagnosis were concentrated in the metropolitan areas of São Paulo and Rio de Janeiro (Figure 2). A total of 304 (93.8%) respondents reported social distancing after mpox diagnosis. Compared to those with no diagnosis, participants reporting an mpox diagnosis were younger (age median 34, IQR 30-39.2 vs median 37, IQR 31-44 years; *P*<.001), more frequently cisgender men (314/324, 96.2% vs 5371/5912, 90.8%; *P*=.02), gay (292/324, 90.1% vs 4905/5912, 83%; *P*<.001), Black (52/324, 16% vs 611/5912, 10.3%; *P*=.008), and living in a state capital city (239/324, 73.8% vs 3978/5912, 67.3%; *P*=.003) (Table 1).

Among participants reporting an mpox diagnosis (n=324), 318 (98.1%) reported lesions, 178 (56%) had local pain, and 316 (99.4%) sought health facilities for diagnosis. The most common lesion sites were genital region (n=155, 48.7%), face (n=128, 40.3%), and anal region (n=118, 37.1%) (Table S4 in Multimedia Appendix 1). Sites with the highest mean numbers of lesions were anal region (4.4, SD 4.3), back (3.7, SD 4.4), genital region (3.2, SD 4.3), and arms (3.2, SD 3.1) (Table S5 in Multimedia Appendix 1). Mpox symptoms were reported by 95.4% (n=309/324) of the participants who reported mpox diagnosis. Among them, 96.8% (n=299/309) attended medical facilities to investigate these symptoms (Table 2), of which the most frequent were headache (n=211, 65.1%) and asthenia (n=205, 63.3%) (Table S6 in Multimedia Appendix 1).

Among participants with no mpox diagnosis, 4974 (84.1%) were aware of mpox lesions, 288 (4.9%) had a suspicious mpox lesion, and 4009 (67.8%) reported potential mpox symptoms. Among individuals with no diagnosis, only 54.9% (n=158/288) and 34.9% (n=1399/4009) attended a health facility to investigate a lesion or any symptoms, respectively (Table 2).

Participants with an mpox diagnosis compared to those with no diagnosis more frequently reported sex partners with suspicious or confirmed mpox (112/324, 34.6% vs 206/5912, 3.5%; *P*<.001), sex with a cisgender man (320/324, 98.8% vs 5132/5912, 86.8%; *P*<.001), more sexual partners (median 10, IQR 5-20 vs median 4, IQR 1-10; *P*<.001), and insertive or receptive anal or oral sex, including condomless sex (*P*<.001 for all) (Table 3). Binge drinking before or during sex (115/324, 35.5% vs 1565/5912, 26.5%; *P*<.001), chemsex (118/324, 36.4% vs 1429/5912, 24.2%; *P*<.001), and any illicit drug use (160/324, 49.4% vs 2354/5912, 39.8%; *P*<.001) were more frequent among those self-reporting an mpox diagnosis. Changes in sexual behavior after the outbreak onset were reported frequently regardless of mpox diagnosis but were higher among participants with a diagnosis (186/324, 57.4% vs 2878/5912, 48.7%; *P*=.002) (Table 4).

**Table 2:** Mpox awareness, knowledge, symptoms, and possible contact according to self-reported mpox diagnosis.

Characteristics			No (n=5912)		Yes (n=324)		Total (N=6236)		*P* value	
Mpox awareness, n (%)			5724 (96.8)		320 (98.8)		6044 (96.9)		.048	
Knowledge of mpox lesions, n (%)			4974 (84.1)		324 (100)		5298 (85.0)		<.001	
**Mpox lesions**										
	Mpox suspicious lesion, n (%)	288 (4.9)		318 (98.1)		606 (9.7)		<.001		
	Local pain (n=606), n (%)	78 (27.1)		178 (56.0)		256 (42.2)		<.001		
	Pain level (n=256), median (IQR)	40 (19-70.8)		75 (49-89.8)		67 (30-84.2)		<.001		
	Attended health facility to investigate lesions (n=606), n (%)	158 (54.9)		316 (99.4)		474 (78.2)		<.001		
**Health facility (n=473), n (%)**										.14
	Public health care	85 (53.8)		192 (61.0)		280 (58.6)				
	Private health services	73 (46.2)		123 (39.0)		196 (41.4)				
Any mpox symptoms, n (%)			4009 (67.8)		309 (95.4)		4318 (69.2)		<.001	
Attended medical service to investigate symptoms (n=4318), n (%)			1399 (34.9)		299 (96.8)		1698 (39.3)		<.001	
**Contact with person with suspicious or confirmed mpox, n (%)**										
	Any	421 (7.1)		112 (34.6)		533 (8.5)		<.001		
	Sex partner	206 (3.5)		112 (34.6)		318 (5.1)		<.001		
**Traveling history since June 2022** **, n (%)**										
	Within Brazil	2459 (41.6)		150 (46.3)		2609 (41.8)		.09		
	Abroad	702 (11.9)		37 (11.4)		739 (11.9)		.80		
**Mpox vaccination, n (%)**										.18
	No	5866 (99.2)		323 (99.7)		6189 (99.2)				
	1 dose	40 (0.7)		0 (0)		40 (0.6)				
	2 doses	6 (0.1)		1 (0.3)		7 (0.1)				
**Country of mpox vaccination, n (%)**										.23
	Canada	11 (24.4)		1 (100)		12 (26.1)				
	United States	25 (55.6)		0 (0)		25 (54.3)				
	France, Italy, or Spain	9 (20.0)		0 (0)		9 (19.6)				

**Table 3:** Sexual behavior and substance use according to self-reported mpox diagnosis.

Characteristics			No (n=5912)		Yes (n=324)		Total (N=6236)		*P* value
**Sex partners, n (%)**									
	Any gender	5330 (90.2)		323 (99.7)		5653 (90.7)		<.001	
	Cisgender man	5132 (86.8)		320 (98.8)		5452 (87.4)		<.001	
	Transgender man	108 (1.8)		5 (1.5)		113 (1.8)		.71	
	Cisgender woman	271 (4.6)		11 (3.4)		282 (4.5)		.32	
	Transgender man	43 (0.7)		2 (0.6)		45 (0.7)		.82	
	Travesti^a^	41 (0.7)		3 (0.9)		44 (0.7)		.63	
	Nonbinary	200 (3.4)		11 (3.4)		211 (3.4)		.99	
	Other gender	51 (0.9)		1 (0.3)		52 (0.8)		.29	
**Number of sex partners (n=5121)**									
	Median (IQR)	4 (1-10)		10 (5-20)		4 (1-10)		<.001	
	0, n (%)	475 (9.7)		1 (0.4)		476 (9.3)		<.001	
	1, n (%)	976 (20)		14 (5.6)		990 (19.3)			
	2-5, n (%)	1716 (35.2)		54 (21.7)		1770 (34.6)			
	6-10, n (%)	915 (18.8)		69 (27.7)		984 (19.2)			
	>10, n (%)	790 (16.2)		111 (44.6)		901 (17.6)			
Steady sex partners, n (%)			2452 (50.3)		121 (48.6)		2573 (50.2)		.59
Only 1 steady sex partner, n (%)			1716 (70.4)		60 (51.3)		1776 (69.5)		<.001
**Sex practices** **(n=5193), n (%)**									
	Insertive vaginal sex	278 (5.6)		10 (4.0)		288 (5.5)		.28	
	Receptive vaginal sex	157 (3.2)		2 (0.8)		159 (3.1)		.03	
	Insertive anal sex	2996 (60.6)		214 (85.9)		3210 (61.8)		<.001	
	Receptive anal sex	2874 (58.1)		187 (75.1)		3061 (58.9)		<.001	
	Insertive oral sex	3760 (76.1)		218 (87.6)		3978 (76.6)		<.001	
	Receptive oral sex	3662 (74.1)		220 (88.4)		3882 (74.8)		<.001	
**Condomless sex (n=5193), n (%)**									
	Any	3535 (72.9)		215 (87.4)		3750 (73.6)		<.001	
	Condomless insertive vaginal sex	172 (3.5)		3 (1.2)		175 (3.4)		.05	
	Condomless receptive vaginal sex	112 (2.3)		2 (0.8)		114 (2.2)		.12	
	Condomless insertive anal sex	2212 (44.7)		180 (72.3)		2392 (46.1)		<.001	
	Condomless receptive anal sex	2126 (43.0)		162 (65.1)		2288 (44.1)		<.001	
	Condomless insertive oral sex	1957 (39.6)		56 (22.5)		2013 (38.8)		<.001	
	Condomless receptive oral sex	2884 (58.3)		195 (78.3)		3079 (59.3)		<.001	
Transactional sex (n=5064), n (%)			175 (3.6)		19 (7.9)		194 (3.8)		<.001
Frequented sex venues, n (%)			2173 (36.8)		206 (63.6)		2379 (38.1)		<.001
**Substance use, n (%)**									
	Binge drinking	3465 (58.6)		200 (61.7)		3665 (58.8)		.27	
	Binge drinking before or during sex	1565 (26.5)		115 (35.5)		1680 (26.9)		<.001	
	Any illicit substance use	2354 (39.8)		160 (49.4)		2514 (40.3)		<.001	
	Cocaine	640 (10.8)		76 (23.5)		716 (11.5)		<.001	
	Marijuana	1770 (29.9)		116 (35.8)		1886 (30.2)		.02	
	Ecstasy	631 (10.7)		51 (15.7)		682 (10.9)		.004	
	Ketamine	324 (5.5)		44 (13.6)		368 (5.9)		<.001	
	GHB^b^	233 (3.9)		31 (9.6)		264 (4.2)		<.001	
	Poppers	566 (9.6)		58 (17.9)		624 (10.0)		<.001	
	Chemsex	1429 (24.2)		118 (36.4)		1547 (24.8)		<.001	

^a^Female gender construction, which is identified in social, family, cultural, and interpersonal life through this identity.

^b^GHB: gamma-hydroxybutyric acid.

**Table 4:** Changes in sexual behavior according to self-reported mpox diagnosis.

Characteristics	No (n=5912), n (%)	Yes (n=324), n (%)	Total (N=6236), n (%)	*P* value
Any change	2878 (48.7)	186 (57.4)	3064 (49.1)	.002
Reduced number of sex partners	2226 (37.7)	149 (46.0)	2375 (38.1)	.003
Avoided sex parties	1596 (27.0)	122 (37.7)	1718 (27.5)	<.001
Avoided bars and clubs	1050 (17.8)	70 (21.6)	1120 (18.0)	.08
Inspected partners for mpox lesions	860 (14.5)	66 (20.4)	926 (14.8)	.004
Asked partners about mpox symptoms	749 (12.7)	50 (15.4)	799 (12.8)	.15
Anal sex only with condoms	742 (12.6)	50 (15.4)	792 (12.7)	.13
Sex abstinence or avoided any type of sex	644 (10.9)	36 (11.1)	680 (10.9)	.90
Avoided kissing	409 (6.9)	26 (8.0)	435 (7.0)	.45
Web-based sex	382 (6.5)	12 (3.7)	394 (6.3)	.047
Washed bed linen and other (eg, towels) after sex	284 (4.8)	26 (8.0)	310 (5.0)	.009
Self-masturbation with partner in the space without touching him or her or they	90 (1.5)	2 (0.6)	92 (1.5)	.19
Wear face masks during sex	79 (1.3)	5 (1.5)	84 (1.3)	.75
Dated people only using clothing	80 (1.4)	2 (0.6)	82 (1.3)	.26
Oral sex with condoms only	63 (1.1)	2 (0.6)	65 (1.0)	.44
Used gloves for anal fisting or fingering	20 (0.3)	1 (0.3)	21 (0.3)	.93

HIV prevalence (122/324, 37.7% vs 1353/5912, 22.9%; *P*<.001), hepatitis C (ever tested positive; 23/324, 7.1% vs 238/5912, 4%; *P*=.007), and any diagnoses of sexually transmitted infections (STIs) (81/324, 25% vs 628/5912, 10.6%; *P*<.001) differed by mpox diagnosis report (Table 5). Among HIV-negative or unknown individuals, HIV testing in the prior 3 months (234/316, 74.1% vs 2335/5545, 42.1%; *P*<.001) and current PrEP use (96/201, 47.8% vs 1106/4537, 24.4%; *P*<.001) were higher among those reporting an mpox diagnosis (Table 5).

Most participants were willing to receive an mpox vaccine (n=5008, 95.1%). When asked about mpox research studies, 74.1% (n=4621) responded that they would participate in prevention vaccine trials, 69.2% (n=4288) in prevention drug trials, and 68.7% (n=4263) in therapeutic trials. In addition, 92% (n=5735) reported frequent participation in vaccination campaigns, with 95.8% (n=4734/4946) coverage of at least 3 doses of the COVID-19 vaccine. Nevertheless, only 47 (0.7%) participants received an mpox vaccine, which is not available in Brazil (Table 2).

Overall, 5258 (84.4%) participants agreed or strongly agreed with the statement: “LGBTQIA+ individuals are being discriminated and stigmatized due to mpox.” Median score of internalized LGBTQIA+ phobia measured by the adapted RHS scale was lower among participants reporting mpox compared to those with no diagnosis (6, IQR 2-11 vs 7, IQR 2-12; *P*<.001).

**Table 5:** HIV testing, treatment, and prevention, and other sexually transmitted infections according to self-reported mpox diagnosis.

Characteristics			No (n=5912), n (%)		Yes (n=324), n (%)		Total (N=6236), n (%)		*P* value	
**HIV status**										<.001
	Negative	4191 (70.9)		194 (59.9)		4385 (70.3)				
	Positive	1353 (22.9)		122 (37.7)		1475 (23.7)				
	Unknown	367 (6.2)		8 (2.5)		375 (6.0)				
**Last HIV test (n=5861)**										<.001
	≤3 months	2335 (42.1)		234 (74.1)		2569 (43.8)				
	>3-6 months	1039 (18.7)		36 (11.4)		1075 (18.3)				
	>6 months	2171 (39.2)		46 (14.6)		2217 (37.8)				
**Time living with HIV (n=1475)**										.006
	≤6 months	14 (1.0)		5 (4.1)		19 (1.3)				
	>6-12 months	42 (3.1)		1 (0.8)		43 (2.9)				
	>12 months	1297 (95.9)		116 (95.1)		1413 (95.8)				
HIV currently undetectable (n=1475)			1250 (92.4)		110 (90.2)		1360 (92.2)		.38	
Adherence to ART^a^ (n=1473)			821 (60.7)		74 (61.2)		895 (60.8)		.93	
**PrEP^b^ use (n=4738)**										<.001
	Never	3132 (69.0)		85 (42.3)		3217 (67.9)				
	Current	1106 (24.4)		96 (47.8)		1202 (25.4)				
	Past	299 (6.6)		20 (10.0)		319 (6.7)				
Complete PrEP adherence (n=1202)			787 (70.6)		73 (75.3)		860 (71.0)		.33	
**STI^c^ diagnoses**										
	Any	628 (10.6)		81 (25.0)		709 (11.4)		<.001		
	Syphilis	442 (7.5)		61 (18.8)		503 (8.1)		<.001		
	Gonorrhea	166 (2.8)		22 (6.8)		188 (3.0)		<.001		
	Chlamydia	94 (1.6)		16 (4.9)		110 (1.8)		<.001		
**Hepatitis B vaccination**										.32
	No vaccination	469 (7.9)		33 (10.2)		502 (8.1)				
	1-2 doses	1395 (23.6)		82 (25.3)		1477 (23.7)				
	3 doses	1739 (29.4)		95 (29.3)		1834 (29.4)				
	Did not remember	2309 (39.1)		114 (35.2)		2423 (38.9)				
Hepatitis C (ever tested positive)			238 (4.0)		23 (7.1)		261 (4.2)		.007	

^a^ART: antiretroviral therapy.

^b^PrEP: pre-exposure prophylaxis.

^c^STI: sexually transmitted infection.

## Discussion

### Principal Findings

This analysis describes the results of a large web-based survey in Brazil conducted during the mpox surge in 2022. Respondents were mostly MSM from metropolitan regions, with a 5.6% prevalence of self-reported mpox diagnosis, which is slightly higher than Brazilian surveillance numbers. This indicates that our survey may have reached populations most affected by mpox by the current outbreak [2,5,6]. There were no reported cases among transgender women, which highlights the low mpox burden in this group, in agreement with Brazilian official data available, disaggregated by gender [8,27]. Our findings point to a high awareness of mpox among SGM, with the internet and television as the most frequent information sources. This underscores the importance of adequate science communication through traditional and innovative means of communication and the need to stop fake news that contributes to misleading information as well as reinforces stigma [28]. Most participants agreed or strongly agreed that LGBTQIA+ individuals are being discriminated against and stigmatized due to mpox. To our knowledge, this is the first study to describe mpox awareness, willingness to vaccinate for mpox, and self-reported prevalence of mpox in Latin America.

### Willingness to Use Mpox Vaccine

Willingness to use mpox vaccine and to follow social isolation if required were high, suggesting an adequate understanding of the importance of public health measures to successfully mitigate the impact of the mpox outbreak among SGM. While countries in North America and Western Europe initiated mpox vaccination of the most affected population (MSM) by mid-2022, mpox vaccination in Brazil only started in March 2023. Due to the low number of vaccine units available, vaccination in the country is restricted to very few groups such as people living with HIV, health professionals working with *Orthopoxvirus* (pre-exposure vaccination), and those who had contact with fluids and secretions of persons with suspected mpox (postexposure vaccination) [29].

### Participants’ Characteristics According to Self-Reported Mpox Diagnosis

In our study, individuals with self-reported mpox diagnosis more frequently referred to mucocutaneous, genital or anal lesions, coinfection with hepatitis C, and local pain. These clinical characteristics are in agreement with global and national data [2,6,30]. Local pain was the most frequent reason for hospitalization in a cohort in Rio de Janeiro State, Brazil [6]. This is critical in the context of poor outcomes related to pain control according to gender, sexual orientation, and race, which might deepen mpox-related stigma and increase the gap between the most vulnerable populations and health services [31,32].

Among respondents with no mpox diagnosis, a substantial proportion did not seek health care assistance despite reporting mpox suspicious lesions or symptoms. Avoidance of health services is common among SGM and directly linked to stigma, discrimination, and structural barriers faced by these groups [33,34]. This might lead to the underreporting of mpox cases in highly discriminatory settings, such as Brazil. In addition, participants with a self-reported mpox diagnosis also reported following a higher frequency of health measures (eg, HIV care and testing, STI diagnosis, and PrEP use) and lower rates of internalized LGBTQIA+ phobia. This might indicate that individuals who sought an mpox diagnosis most commonly have access to health services in general. Furthermore, these participants might have previous links with gender-competent health care services and more access to health information, all potentially related to a lower perceived gender and sexual orientation–based discrimination.

Although participants with an mpox diagnosis reported a higher number of sexual partners, binge drinking before sex, chemsex, substance use, and higher frequency to sex venues, they also adopted more changes in sexual behavior after the mpox outbreak, possibly due to higher awareness and feelings of fear and hopelessness related to mpox. This finding is in agreement with previous surveys conducted in the United States among the most vulnerable populations [16,35]. Moreover, most individuals with an mpox diagnosis in our survey also reported sex with other cisgender men, reinforcing the role of highly interconnected and dense sexual networks in the mpox transmission dynamics [36]. Even among participants who self-reported an mpox diagnosis, only a small proportion reported a partner with suspected or confirmed mpox. Although this observation might be related to underdiagnosed cases, it raises concerns about the potential role of subclinical infections in mpox transmission dynamics, while it also reflects the well-known challenges in STI network mapping [37-39].

People living with HIV enrolled in the current analysis reported low ART adherence (895/1473, 60.8%). Regardless of mpox diagnosis, this reinforces the urgent need to better monitor the HIV care continuum. In the context of mpox, advanced immunosuppression and poor ART adherence have been associated with worse mpox clinical outcomes, including mpox-related hospitalization and death [40]. The high rates of other STIs occurring concomitantly with mpox highlight the importance of a comprehensive screening at mpox assessment, in accordance with the well-established rationale that 1 STI diagnosis implies a higher likelihood of coinfection with an additional one [41].

### Limitations

Our study has some limitations. First, the cross-sectional study design hinders identifying causal associations. In addition, all responses were self-reported, thus introducing the possibility of recall, response, or social desirability bias. Moreover, our sample constitutes a highly educated, middle or high socioeconomic status subset of Brazilian persons with access to a device compatible with GSN apps, although cellphones and internet connection have been shown to be widely available in all socioeconomic strata in Brazil [42]. Transgender women are often a highly vulnerable group in Brazil with lower access to the internet, which may have impacted their recruitment using web-based strategies as previously observed [17].

### Conclusions

Our results point to high mpox knowledge and willingness to vaccinate for mpox among SGM in Brazil. Participants self-reporting mpox diagnosis more frequently reported to be living with HIV, STI diagnosis, and current PrEP use. Our findings highlight the importance of an mpox assessment that includes comprehensive sexual health screenings. Efforts to decrease stigma related to mpox among SGM is necessary to avoid mpox underdiagnosis.

## Appendix

Multimedia Appendix 1 Supplementary tables.

## Abbreviations

ART: antiretroviral therapy
LGBTQIA+: Lesbian, gay, bisexual, transgender, queer, intersex, asexual, plus
MSM: men who have sex with men
MW: minimum wage
PrEP: pre-exposure prophylaxis
RHS: Reactions to Homosexuality Scale
SGM: sexual and gender minorities
STI: sexually transmitted infection

## Data Availability

The study’s final deidentified data set and dictionary will be made available with the publication upon reasonable request. A proposal should be submitted to the corresponding author’s email, who will evaluate and approve the request. No other additional documents will be available.
