# Supplementary material for: Evaluation of Mpox Knowledge, Stigma, and Willingness to Vaccinate for Mpox: Cross-Sectional Web-Based Survey Among Sexual and Gender Minorities
Source: JMIR Public Health Surveill. 2023 Jul 17;9:e46489. doi: 10.2196/46489 (PMC10411424; doi:10.2196/46489)
Supplement: Multimedia Appendix 1 [file publichealth_v9i1e46489_app1.docx]

**Supplementary Table 1. Adaptation of The Reactions to Homosexuality Scale items**

|  | **The Reactions to Homosexuality Scale original items** | **The Reactions to Homosexuality Scale adapted items** |
| --- | --- | --- |
| Item 1 | I feel comfortable in gay bars. | I feel comfortable in LBGTQIA+ bars. |
| Item 2 | Social situations with gay men make me feel uncomfortable. | Social situations with LBGTQIA+ persons make me feel uncomfortable. |
| Item 3 | I feel comfortable being seen in public with an obviously gay person. | I feel comfortable being seen in public with an obviously LBGTQIA+ person. |
| Item 4 | I feel comfortable discussing homosexuality in a public situation. | I feel comfortable discussing my sexuality in a public situation. |
| Item 5 | I feel comfortable being a homosexual man. | I feel comfortable being a LBGTQIA+ person. |
| Item 6 | Homosexuality is as natural as heterosexuality. | Homosexuality is as natural as heterosexuality. |
| Item 7 | Even if I could change my sexual orientation, I wouldn’t. | Even if I could change my sexual orientation, I wouldn’t. |

**Supplementary Table 2. Where participants heard about mpox.**

|  | Total |
| --- | --- |
|  | 6236 (%) |
| Internet | 4793 (76.9) |
| Television | 4519 (72.5) |
| Google | 3176 (50.9) |
| Grindr | 896 (14.4) |
| Radio | 853 (13.7) |
| Scruff | 423 (6.8) |
| Hornet | 147 (2.4) |
| Other | 1244 (19.9) |
| Did not remember | 34 (0.5) |

**Supplementary Table 3. Self-reported mpox diagnosis according to Brazilian State.**

|  | **Self-reported mpox diagnosis** | | |
| --- | --- | --- | --- |
|  | No | Yes | Total |
|  | 5912 (%) | 324 (%) | 6236 (%) |
| **North** |  |  |  |
| Acre | 4 (0.1) | 0 (0) | 4 (0.1) |
| Amapá | 4 (0.1) | 0 (0) | 4 (0.1) |
| Amazonas | 40 (0.7) | 0 (0) | 40 (0.6) |
| Pará | 60 (1) | 3 (0.9) | 63 (1) |
| Rondônia | 9 (0.2) | 0 (0) | 9 (0.1) |
| Roraima | 8 (0.1) | 0 (0) | 8 (0.1) |
| Tocantins | 9 (0.2) | 0 (0) | 9 (0.1) |
|  |  |  |  |
| **Northeast** |  |  |  |
| Alagoas | 24 (0.4) | 1 (0.3) | 25 (0.4) |
| Bahia | 221 (3.7) | 4 (1.2) | 225 (3.6) |
| Ceará | 151 (2.6) | 9 (2.8) | 160 (2.6) |
| Maranhão | 31 (0.5) | 0 (0) | 31 (0.5) |
| Paraíba | 34 (0.6) | 1 (0.3) | 35 (0.6) |
| Pernambuco | 140 (2.4) | 9 (2.8) | 149 (2.4) |
| Piauí | 26 (0.4) | 1 (0.3) | 27 (0.4) |
| Rio Grande do Norte | 57 (1) | 4 (1.2) | 61 (1) |
| Sergipe | 25 (0.4) | 0 (0) | 25 (0.4) |
|  |  |  |  |
| **Central west** |  |  |  |
| Distrito Federal | 238 (4) | 16 (4.9) | 254 (4.1) |
| Goiás | 105 (1.8) | 9 (2.8) | 114 (1.8) |
| Mato Grosso | 37 (0.6) | 4 (1.2) | 41 (0.7) |
| Mato Grosso do Sul | 31 (0.5) | 7 (2.2) | 38 (0.6) |
|  |  |  |  |
| **Southeast** |  |  |  |
| Espírito Santo | 64 (1.1) | 3 (0.9) | 67 (1.1) |
| Minas Gerais | 435 (7.4) | 23 (7.1) | 458 (7.3) |
| Rio de Janeiro | 1245 (21.1) | 51 (15.7) | 1296 (20.8) |
| São Paulo | 2294 (38.8) | 144 (44.4) | 2438 (39.1) |
|  |  |  |  |
| **South** |  |  |  |
| Paraná | 220 (3.7) | 13 (4) | 233 (3.7) |
| Rio Grande do Sul | 220 (3.7) | 16 (4.9) | 236 (3.8) |
| Santa Catarina | 180 (3) | 6 (1.9) | 186 (3) |

**Supplementary Table 4. Suspicious lesions of mpox according to self-reported mpox diagnosis.**

|  | **Self-reported mpox diagnosis** | | |  |
| --- | --- | --- | --- | --- |
|  | No | Yes | Total | p-value |
|  | 5912 (%) | 324 (%) | 6236 (%) |  |
| Genital | 85 (29.5) | 155 (48.7) | 240 (39.6) | < .001 |
| Face | 57 (19.8) | 128 (40.3) | 185 (30.5) | < .001 |
| Arms | 70 (24.3) | 110 (34.6) | 180 (29.7) | .006 |
| Anus | 56 (19.4) | 118 (37.1) | 174 (28.7) | < .001 |
| Back | 60 (20.8) | 113 (35.5) | 173 (28.5) | < .001 |
| Thighs | 57 (19.8) | 72 (22.6) | 129 (21.3) | .39 |
| Abdomen | 43 (14.9) | 71 (22.3) | 114 (18.8) | .02 |
| Chest | 36 (12.5) | 75 (23.6) | 111 (18.3) | < .001 |
| Feet | 22 (7.6) | 76 (23.9) | 98 (16.2) | < .001 |
| Hands | 19 (6.6) | 69 (21.7) | 88 (14.5) | < .001 |
| Forearms | 38 (13.2) | 46 (14.5) | 84 (13.9) | 0.65 |
| Mouth | 26 (9.0) | 30 (9.4) | 56 (9.2) | 0.86 |
| Nose | 6 (2.1) | 28 (8.8) | 34 (5.6) | < .001 |
| Other | 40 (13.9) | 55 (17.3) | 95 (15.7) | 0.25 |

**Supplementary Table 5. Mean number of mpox lesions among participants who self-reported mpox diagnosis (N=324).**

|  | Mean (SD) |
| --- | --- |
|  |  |
| Anus | 4.4 (4.3) |
| Back | 3.7 (4.4) |
| Genital | 3.2 (4.3) |
| Arms | 3.2 (3.1) |
| Chest | 3.0 (3.8) |
| Forearms | 2.9 (2.1) |
| Abdomen | 2.8 (2.7) |
| Thighs | 2.8 (2.4) |
| Face | 2.5 (2) |
| Feet | 2.3 (2.4) |
| Hands | 2.2 (1.3) |
| Mouth | 2.0 (1.8) |
| Nose | 1.6 (0.9) |

SD: standard deviation

**Supplementary Table 6. Symptoms according to self-reported mpox diagnosis.**

|  | **Self-reported mpox diagnosis** | | |  |
| --- | --- | --- | --- | --- |
|  | No | Yes | Total | p-value |
|  | 5912 | 324 | 6236 |  |
| Headache | 2695 (45.6) | 211 (65.1) | 2906 (46.6) | < .001 |
| Sore throat | 2105 (35.6) | 155 (47.8) | 2260 (36.2) | < .001 |
| Asthenia | 1659 (28.1) | 205 (63.3) | 1864 (29.9) | < .001 |
| Myalgia | 1605 (27.1) | 174 (53.7) | 1779 (28.5) | < .001 |
| Diarrhea | 1226 (20.7) | 95 (29.3) | 1321 (21.2) | < .001 |
| Arthralgia | 576 (9.7) | 65 (20.1) | 641 (10.3) | < .001 |
| Vomiting | 403 (6.8) | 40 (12.3) | 443 (7.1) | < .001 |
| Dizziness | 372 (6.3) | 43 (13.3) | 415 (6.7) | < .001 |
| Constipation | 363 (6.1) | 36 (11.1) | 399 (6.4) | < .001 |
| Dyschezia | 245 (4.1) | 100 (30.9) | 345 (5.5) | < .001 |
| Inguinal adenopathy | 148 (2.5) | 181 (55.9) | 329 (5.3) | < .001 |
| Chest pain | 273 (4.6) | 25 (7.7) | 298 (4.8) | .01 |
| Cervical adenopathy | 211 (3.6) | 74 (22.8) | 285 (4.6) | < .001 |
| Ophthalmological complaints | 248 (4.2) | 23 (7.1) | 271 (4.3) | .01 |
| Anal ulcer | 139 (2.4) | 75 (23.1) | 214 (3.4) | < .001 |
| Anal pain | 129 (2.2) | 76 (23.5) | 205 (3.3) | < .001 |
| Genital edema | 81 (1.4) | 93 (28.7) | 174 (2.8) | < .001 |
| Dysuria | 135 (2.3) | 25 (7.7) | 160 (2.6) | < .001 |
| Axillary adenopathy | 97 (1.6) | 30 (9.3) | 127 (2.0) | < .001 |
| Other adenopathy | 29 (0.5) | 17 (5.2) | 46 (0.7) | < .001 |
